# The Imaramagambo Onchocerciasis Focus in Southwestern Uganda: Interruption of Transmission after Disappearance of the Vector *Simulium neavei* and Its Associated Freshwater Crabs

**DOI:** 10.4269/ajtmh.16-0181

**Published:** 2016-08-03

**Authors:** Moses N. Katabarwa, James Katamanywa, Thomson Lakwo, Peace Habomugisha, Edson Byamukama, David Oguttu, Christine Nahabwe, Monica Ngabirano, Ephraim Tukesiga, Annet Khainza, Edridah Tukahebwa, Thomas R. Unnasch, Frank O. Richards, Rolf Garms

**Affiliations:** ^1^ Health Programs, The Carter Center, Atlanta, Georgia; ^2^ Health Services, Kabarole District, Fort Portal, Uganda; ^3^ Vector Control Division, Ministry of Health, Kampala, Uganda; ^4^ Health Programs, The Carter Center, Kampala, Uganda; ^5^ University of South Florida, Global Health, Tampa, Florida; ^6^ Tropical Medicine Department, Bernhard Nocht Institute of Tropical Medicine, Hamburg, Germany

## Abstract

It was not until early 1990s that, when the Imaramagambo focus of southwest Uganda was mapped, mass treatment with a single annual dose of ivermectin for onchocerciaisis control commenced. However, comprehensive investigations on its transmission were launched after a nationwide policy for onchocerciasis elimination in 2007. Entomological surveys throughout the focus from 2007 to 2015 have yielded few or no freshwater crabs (*Potamonautes aloysiisabaudiae*), which serve as the obligate phoretic host of the larvae and pupae of the vector *Simulium neavei*. No *S*. *neavei* flies have been observed or collected since 2007. Skin snips (microscopy) from 294 individuals in 2008 were negative for skin microfilariae, and of the 462 persons analyzed by polymerase chain reaction skin snip poolscreen in 2009, only five (1.08%) persons were indicated as infected with onchocerciasis. All five of the positive persons were at least 40 years old. Serosurvey results showed negative exposure among 3,332 children in 2012 and 3,108 children in 2015. Both were within the upper bound of the 95% confidence interval of the prevalence estimate of 0.06%, which confirmed the elimination of onchocerciasis. Treatment coverage in Imaramagambo was generally poor, and transmission interruption of onchocerciasis could not be attributed solely to annual mass treatment with ivermectin. There was sufficient evidence to believe that the possible disappearance of the *S*. *neavei* flies, presumed to have been the main vector, may have hastened the demise of onchocerciasis in this focus.

## Introduction

Before 1970, the focus of Imaramagambo was still a sparsely populated jungle where onchocerciasis was largely unknown, as exemplified by its absence on the list of onchocerciasis foci in Uganda.^1^–^3^ However, as more people migrated to the area to exploit its fertile agricultural land, onchocerciasis began to emerge as a public health problem. The East Ankole Diocese (Mbarara, Uganda), through its eye care program, launched onchocerciasis control activities in the Imaramagambo focus with financial assistance from Christoffel Blindenmission (CBM) in 1993. In 1996, the Ministry of Health took over this program with support from the African Program for Onchocerciasis Control (APOC). By then, onchocerciasis constituted 17.4% of the total eye disease burden in Imaramagambo.^4^

The Imaramagambo onchocerciasis focus stretches from southwest of Lake Edward, from the Kigezi game reserve south toward the Bwindi impenetrable forest^5^,^6^ and Kashoya-Kitomi, east of Lake George.^7^ The focus includes portions of the Imaramagambo and Kalinzu natural forests. In an unpublished working paper by Angus McCrae, it was generally stated that *S*. *neavei* transmits onchocerciasis in a continuous range along the eastern slopes of the western Rift Valley escarpment, draining into Lakes Edward, George, and Albert, where the *Simulium* flies were universally prevalent along rivers with a gallery forest ecology.^8^ In a letter dated October 14, 1966, Angus McCrae, former senior entomologist, Ministry of Health Uganda, explicitly wrote, “In the upper Nchwera in the Kalinzu Forest, the crabs appeared to be *P*. *aloysiisabaudiae* of the Rwenzori type, and not of the Ishasha system type with scarlet intersegmental membranes of the chelae.”

Generally, it was assumed that *S*. *neavei* was the vector for onchocerciasis in Imaramagambo onchocerciasis focus. The larvae of this vector develop in an obligate phoretic association with the freshwater crab, *Potamonautes aloysiisabaudiae*. The Imaramagambo onchocerciasis focus as it is known today is separated from Kashoya-Kitomi forest (399 km^2^) by the 2-km wide Rubare ridge, whose altitude ranges from 1,493 to 1,574 m. All the main rivers flow toward Lake Edward and have their catchments in cultivated areas either in large tea plantations bordering the forest reserves or east of the forests. Usually, biting densities of *S*. *neavei* occur with a narrow range, extending up to 4 km from the forest edge.^9^

**Figure 1.**
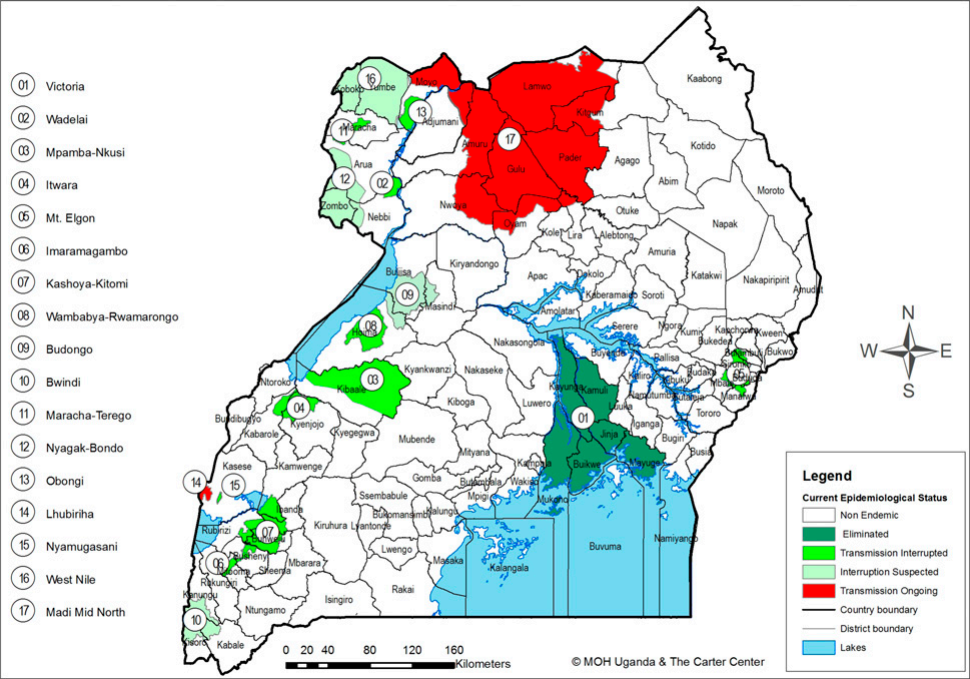
Map of Uganda showing the onchocerciasis foci of Uganda and progress toward onchocerciasis elimination.

Uganda has 17 onchocerciasis foci, spread in central, eastern, northern, western, and southwestern regions (Figure 1

). *Simulium damnosum* s.l. and *S*. *neavei* are the main vectors of onchocerciasis. The world's inventory of blackflies indicates a list of eight cytoforms of *S*. *damnosum* s.l. listed for Uganda: *S*. *damnosum* s.str., *Simulium sirbanum*, *Simulium kilibanum*, ‘Nkusi’, ‘Sebwe’, ‘Kagera’, ‘Kaku’, and *Simulium pandanophilum*.^10^ *Simulium damnosum* s.l. was the main vector in Victoria Nile focus of central Uganda, which was eliminated in the 1970s. Other areas affected by *S*. *damnosum* s.l. include Lhubiriha focus of western Uganda in Kasese District and Madi-Mid North focus in the north. The area affected by *S. damnosum* s.l. has a population of at least 4 million people, whereas *S. neavei* has been transmitting onchocerciasis among a population of about 1.5 million people. *Simulium neavei* predominantly occurs at a higher altitude than *S. damnosum* s.l. and is responsible for onchocerciasis transmission in the eastern, northwestern, southwestern, and western regions of Uganda.

In 2007, the government of Uganda, based on the available evidence that twice-yearly treatment can eliminate onchocerciasis within six and a half years, launched a nationwide onchocerciasis elimination policy.^11^ A twice-yearly strategy was chosen because studies have suggested that annual treatments may require up to 25 years to eliminate the infection.^12^ The national elimination policy had promoted rigorous epidemiological, serological, and entomological assessments to determine the status of onchocerciasis in every endemic area in Uganda after many years of a single annual dose of ivermectin. The objective of the government of Uganda is to eliminate onchocerciasis by 2020.

The Imaramagambo focus is one of the three onchocerciasis foci (Imaramagambo, Mpamba-Nkusi, and Maracha-Terego) where transmission of onchocerciasis was thought to have been eliminated. In 2012, it was ascertained that indeed transmission had been interrupted and annual mass treatment was discontinued in this focus, beginning a 3-year posttreatment surveillance (PTS) period from 2013. Here, we report the results of the entomological and epidemiological surveys conducted since 2007 to ascertain the transmission of onchocerciasis as well as information collected during the course of the 3-year PTS period.

## Materials and Methods

### Study area.

Imaramagambo, located in the southwest, is one of the 17 onchocerciasis foci in Uganda. It lies east of the large blocks of the Imaramagambo and Kalinzu forest reserves in Bushenyi District (currently divided into Bushenyi and Mitooma districts) and Lake Edward (Figure 1). According to Howard,^13^ the forest reserves cover an area of 580 km^2^ (north Imaramagambo 291 km^2^, south Imaramagambo 152 km^2^, and Kalinzu 137 km^2^), and 299 km^2^ of the reserves fall within the area of the Queen Elizabeth National Park, which borders Lake Edward.

### Assessments in the early 1990s.

Surveys conducted by East Ankole Diocese from 1992 to 1993 revealed that onchocerciasis was an important health problem in the Bushenyi District. Unpublished reports stated the prevalence of punctate keratitis as greater than 1%, whereas chronic onchocercal ocular disease (sclerosing keratitis) was rare. In the early 1990s, population assessments using the rapid epidemiological assessment technique indicated a nodule prevalence from 10% to 19% in the south, and up to 40% in the north of the Bushenyi District.^4^ In 1999, an ophthalmologic impact study was carried out on 367 persons aged 10 years and above from seven selected villages.^4^ Although acute onchocerciasis–related lesions were rare, irreversible lesions were still present, and dead microfilariae were found in the eyes of two persons. On the basis of this finding, a strengthened mass treatment with ivermectin was recommended.^4^

### Annual mass treatment.

Annual mass treatment with ivermectin began in 1993 in the East Ankole Diocese with support from CBM; the program continued for 3 years under CBM supervision, after which the Ministry of Health took over the activities. APOC assistance for community-directed treatment with ivermectin activities in this focus commenced in 1999, covering 212 communities east of the Imaramagambo-Kalinzu forest reserve and south of the Kashoya-Kitomi forest reserve. Data on the ultimate treatment goal (UTG) with annual mass treatment with ivermectin was available from 1993 to 2009 (Table 1). The UTG is the sum of all eligible persons for treatment (minus children < 5 years of age) among the total number of people at risk living in an onchocerciasis-endemic area.^14^ After 2009, treatment data were not available, although treatment seemed to have taken place until 2012, when the focus was declared interrupted by the Uganda Onchocerciasis Elimination Expert Advisory Committee (UOEEAC), and the focus was approved for a 3-year PTS phase.^15^

### Entomological assessments.

The distribution of *S. neavei* and that of its phoretic crab host in the Imaramagambo focus were not assessed before interventions were launched in the early 1990s. A short entomological survey carried out in 2007 alluded to the absence of crabs and consequently to the vector (J. Katamanywa, E. Mutuuzi, J. Wamani, J. Kashaija, unpublished report). More detailed follow-up surveys were conducted in 2008–2009 and 2012–2015 to determine whether onchocerciasis transmission was still ongoing in the focus (Figure 1). Scale 1:50,000 maps of Rwenshama (75/4), Rubirizi (76/3), (84/2), and Bushenyi (85/1), published by the Department of Lands and Surveys, Uganda, were used for the surveys. GPS instruments (GARMIN eTrex, Kansas City, KS) were used to determine the location of the study sites. Instruments were set to the map datum 1,960 arc of the Uganda 1:50.000 maps.

### Search for freshwater crabs.

The objective of the surveys was to ascertain whether the freshwater crab *P. aloysiisabaudiae*, which serves as the obligate phoretic host of the larvae and pupae of the vector *S. neavei*, still occurred in the focus. Crabs were caught by means of funnel traps (total funnel length 36 cm, width 25 cm, and entrance 6.3 cm) constructed by one of us (Ephraim Tukesiga). These crab traps have been successfully used to trap crabs in the *S. neavei-*transmitted onchocerciasis foci of Uganda, and are known for their reliable results on the occurrence of crabs and their infestations with immature stages of *S. neavei*.^16^–^18^ The traps were baited with fresh meat and left in the river for 1 hour, several hours, or overnight. The carapace width of all male, immature and mature female crabs caught was measured and recorded.^16^ The crabs were returned to the river/stream immediately after examination.

### Search for adult flies.

**Figure 2.**
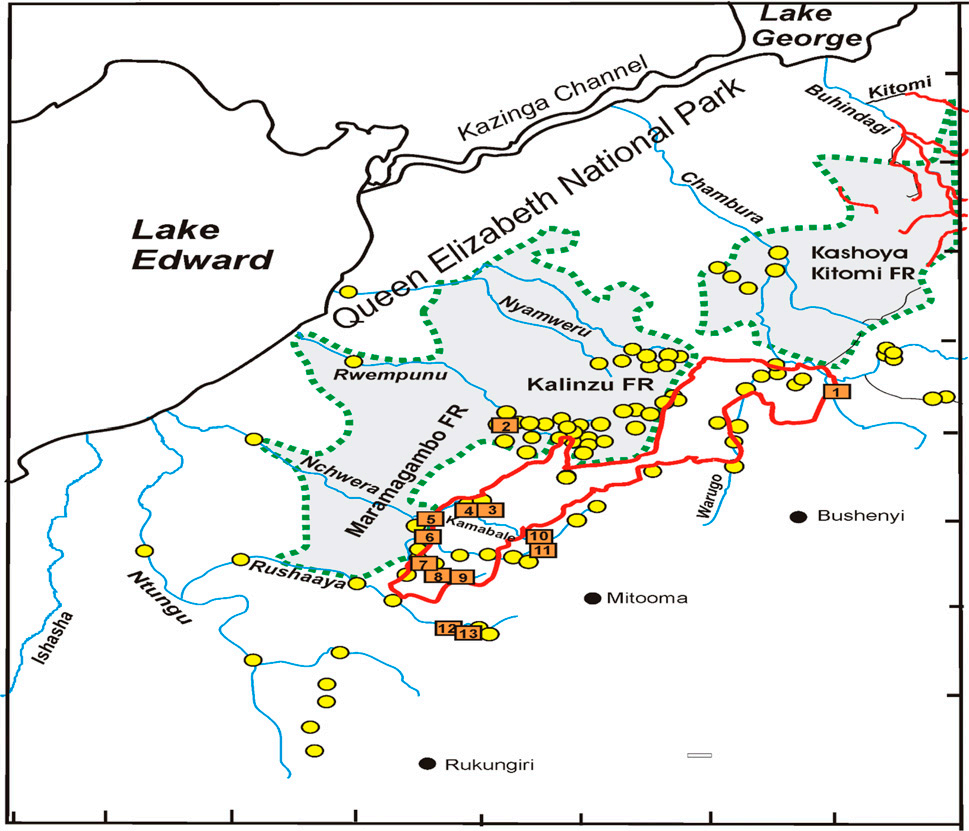
The Imaramagambo onchocerciasis focus in southwestern Uganda in Bushenyi and Mitooma districts associated with the Kalinzu and Maramagambo forest reserves (FR) to the West and the Kashoya-Kitomi FR to the northeast. 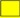 = the sites where few *Potamonautes aloysiisabaudiae* were collected during surveys in 2008, 2009, and 2012 (cf. Table 2); 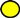 = the sites where there were no crabs (cf. Table 2); 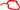 = rivers in the Kashoya-Kitomi focus where *P. aloysiisabaudiae* still occurs; = red line encircles the area where 212 communities were under annual treatment.

Whenever possible, and particularly while allowing the traps to collect crabs, the entomological team collected *Simulium* flies landing upon them. Fly collection was done routinely from January 2012 to February 2015 at five fly collection sites (Figure 2

).

### Surveys in 2007 and 2008.

The surveys in 2007 and 2008 were primarily conducted along the eastern and southern fringes of the forest reserves in the Bushenyi District near the formerly endemic areas. In 2007, 15 sites were visited whereas in 2008, 45 sites in different watercourses in and at the edge of the forest reserves, as well as in open areas east of the forests, were examined for the presence of freshwater crabs. One day was spent on Ntungu River south of the Imaramagambo-Kalinzu forest in the Rukungiri District.

### Assessments 2009.

In 2009, entomological prospections followed all rivers crossing the Queen Elizabeth National Park before they entered Lake Edward. These were done to determine whether freshwater crabs and *S. neavei* had withdrawn to the lower stretches of rivers. Some sites in the Kalinzu forest reserve that had been assessed in 2008 were rechecked in 2009.

### Assessments 2012–2015.

Altogether, 33 sites in different watercourses in and at the edge of the forest reserves and in open areas east and south of the forests were surveyed from January to August for the presence of freshwater crabs. In the northern part of the focus, in the Bushenyi District, the Kyambura system with rivers Kyambura, Warugo, Nyakasolo, and Misimiro were surveyed. Of special interest was the southern part of the focus east of the South Imaramagambo forest reserve, where surveys were conducted in early 2012. A total of 16 sites were visited. In this area, the river systems drain to the west through Kalinzu and north Imaramagambo forest reserves, and the Queen Elizabeth National Park to Lake Edward. The team did not venture deep into the forest reserve as it was risky. This risk was due to little or no human activities coupled with the presence of wild animals and the absence of cleared forest roads, which was further complicated by the onset of the rainy season. Systematic crab trapping and fly collection occurred from January 2012 to February 2015 (Figure 2).

### Parasitological assessment.

In parallel to the entomological studies, a short parasitological survey through skin snips (microscopy) was carried out in 2008 in four villages covering a sample of 294 persons of all ages. The standard protocol for skin snipping was followed, whereby two skin snips were taken from the iliac crest posteriorly for every selected adult.^19^ The skin snips were read under low (×40) magnification after a 12-hour incubation at ambient temperature in normal saline solution.

In 2009, skin snips collected from a total of 462 adults and children aged 5 years and above from six communities (two in the Bushenyi District and four in the Mitooma District) were tested for the presence of parasite DNA by polymerase chain reaction (PCR). Of the individuals examined, 29 were 5–9 years old, 78 were 10–14 years old, 52 were 15–19 years old, 91 were 20–29 years old, 74 were 30–39 years old, and 138 were 40 years old or over.

After transportation to the central laboratory, the skin samples were transferred to phosphate-buffered saline and incubated for 20 minutes at room temperature. The snip was then placed in a small glass homogenizer containing 60 μL of 100 mM NaCl, 10 mM Tris-HCl (pH 8.0), 1 mM ethylenediaminetetraacetic acid, 0.1% sodium dodecyl sulfate, and 10 μg/mL salmon sperm DNA. The sample was then homogenized to break up the tissue as much as possible, and 20 μL of 400 μg/mL proteinase K solution was added to the homogenate. The solution was incubated at 55°C for 1 hour. Of 100 mM dithiothreitol, 8 μL was added to the homogenate, and the sample was incubated at 100°C for 30 minutes. The homogenate was then subjected to three freeze thaw cycles and centrifuged at 13,100 × *g* for 1 minute to pellet the debris. The supernatant was transferred to a new tube, and the DNA in the sample was purified by silica absorption as previously described.^19^ The adsorbed DNA was eluted into 50 μL of molecular grade water, and 5 μL of the elute was used as a template in a PCR targeting the O-150 repeat family of *Onchocerca volvulus*, as described previously.^20^

### Serological assessments, 2009.

**Figure 3.**
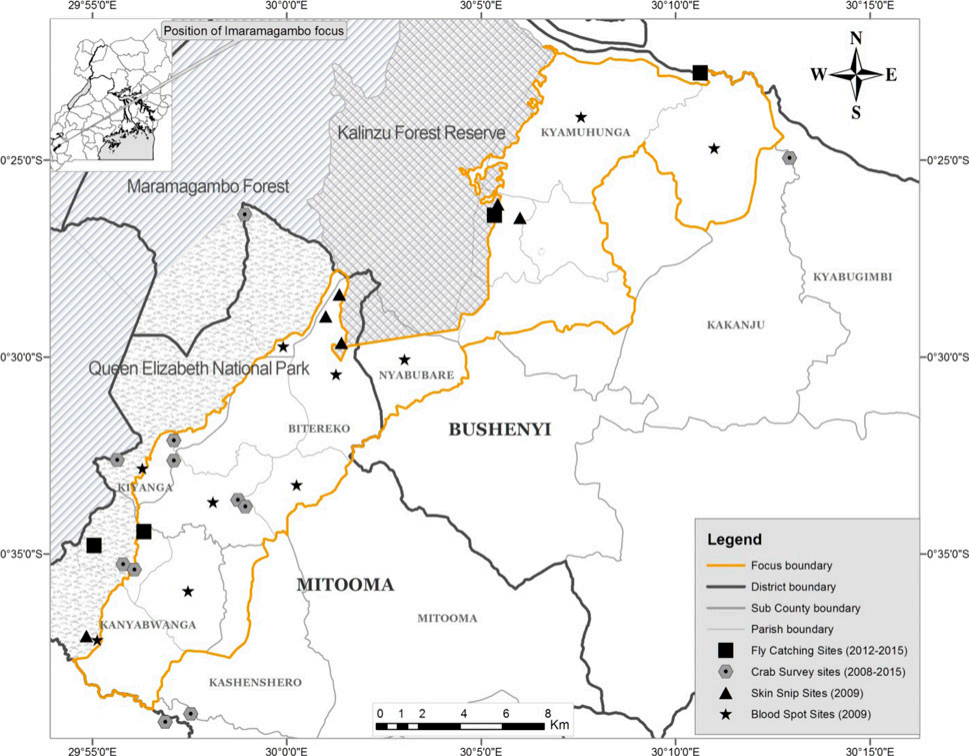
Map of Imaramagambo onchocerciasis focus showing sites where blood spots and skin snips were collected and where crab trapping and fly catching were done.

Random sampling was done based on parishes, each composed of many communities, and 10 of 17 parishes were selected for assessment. Blood spots were collected from a sample of 3,332 children (≥ 1–14 years of age) in 2009 from three parishes (1,117 blood spots) and seven parishes (2,215 blood spots) in the Bushenyi and Mitooma districts, respectively (Figure 3

). The age distribution of the individuals enrolled in the study was as follows: 1–4 years: 1,392 (41.8%); > 4–9 years: 968 (29.1%); and > 9–14 years: 972 (29.2%). During blood spot collection, disposable needles and sterile procedures were applied to collect blood spots on Whatman No. 2 filter paper (Sigma, St. Louis, MO). The blood samples were dried, separated by sheets of paper, and systematically bundled and stored under desiccation in plastic bags in a cooler. On delivery to the laboratory, they were stored at 4°C before being processed for analysis. Sera were eluted from the dried spots and examined for the presence of Ov16 IgG4 antibodies by enzyme-linked immunosorbent assay, as previously described.^21^ To confirm infections in Ov16 putative positive children, skin snips were collected after standard procedures.^22^ Detection of parasite DNA using a PCR assay was chosen to analyze these skin snips as a confirmatory test for Ov16 positivity.^23^

### National guidelines for onchocerciasis elimination.

The Ministry of Health, Uganda, established UOEEAC in 2008 to guide the nationwide River Blindness Elimination Program and provide recommendations for decision making on when and why to stop interventions and move the concerned foci to PTS phase and subsequent elimination of the disease. The national guidelines were based on the 2001 World Health Organization criteria for certification of elimination of onchocerciasis.^24^ The national guidelines include three indicators: 1) parasitological assessments: microfilaria (mf) prevalence in skin snips must be less than 5% in all sampled communities and less than 1% in 90% of sampled communities; 2) serological assessments: infection rates in a sample of 3,000 children must be < 0.1% (to exclude 0.1% antibody prevalence with 95% confidence interval [95% CI]); and 3) entomological assessments: in the *S. neavei* foci, an absence of positive crabs for larvae/pupae of *S. neavei* species in a series of surveys and an absence of *S. neavei* or *S. damnosum* collected in a defined focus over a period of 3 years when the objective is vector elimination.^15^,^25^

### PTS period (2013–2015).

When interruption of transmission was declared in 2012, the focus was moved to the 3-year PTS period. The districts involved (Bushenyi and Mitooma) were officially informed by the Ministry of Health about interruption of transmission of onchocerciasis and halting of all interventions. Advocacy meetings were held at district, subcounty, and community levels with the aim of informing and educating affected communities as to why interventions were halted, and what their responsibilities would be during the PTS period. Community members are expected to report any cases with symptoms of onchocerciasis observed as well as any black flies biting them. During the PTS period, entomological surveillance continued on an annually and quarterly basis. During the last quarter of 2015, in a serological survey, 3,108 blood spots were taken from a sample of children under 10 years of age from the entire focus. Similar procedures were followed as in the 2012 serological survey, but this time only children under 10 years of age were considered.^21^

### Data analysis.

Parasitological data from adults and children and serological data from children were entered and analyzed in Microsoft Excel (Microsoft Corp., Redmond, WA). Where relevant, the data were entered and analyzed in Epi Info (Centers for Disease Control and Prevention, Atlanta, GA) for a χ^2^ test of independence. The entomological data were also entered, analyzed, and graphically illustrated in Microsoft Excel.

### Ethical approval of the study protocols.

The Ministry of Health, Uganda, and Emory Institutional Review Board (11 438) classified the assessment activities as periodical program performance assessment and therefore as non-research. The approved procedure for parasitological, serological, and entomological evaluations are indicated in the national guidelines for verification of elimination of onchocerciasis as standard methods for use in the onchocerciasis elimination program. In parasitological and serological epidemiology studies, consent was obtained from all participants and communities involved. Verbal assent was obtained from the parents of young children. All participating communities were educated about the importance of evaluations, and participants were assured that there would be no repercussions for refusing to participate. The program recruited persons aged 20 years and older as *Simulium* collectors, after first making them aware of the nature of the work, and obtaining informed consent. The collectors were then trained and deployed. They could also opt out of the study at any time without any repercussions if they desired.

## Results

### Treatments.

With the exception of 2009, where 94.4% of the UTG coverage was attained, the treatment coverage in this focus was below 80% from 1993 to 2008. The desired coverage was at least 90% of UTG. No treatment results were provided for 2008, and there are no records to document that treatment was provided after 2009 (Table 1).

### Entomological assessments.

#### Assessments 2007–2008.

The observations in all 15 sites assessment in 2007 and all 45 in 2008 indicated a complete absence of *P. aloysiisabaudiae*, the phoretic host of the immature stages of *S. neavei*, in the focus (Figure 2 and Table 2). Also, no biting *Simulium* flies were observed.

Small crabs of an unknown species were collected in two rivers not suitable for *S. neavei*. Of particular interest was the Nchwera River's (Rwempunu) tributaries, the Ruzoonga, where a single specimen of *P. aloysiisabaudiae* was collected in 2008.

#### Assessments in 2009.

Surveys on the lower stretches of all rivers crossing Queen Elizabeth National Park before entering Lake Edward yielded no crabs; these rivers included the Nyamweru, Rwempunu, and Kaizi Nchwera rivers within the Queen Elizabeth National Park. The same results were obtained on the Ntungu and Rushaaya rivers in the area outside of the park (Figure 2). No crabs were found in the rivers that flow through the Kalinzu forest reserve either.

#### Assessments in 2012.

Crabs caught at 10 sites (1, 3, 4, 6, 7, 8, 9, 10, 12, and 13), altogether during 21 trapping days were negative for immature stages of *S. neavei* (Table 2). Only in the Nyakasolo bridge site, three contained uninfested *P. aloysiisabaudiae* (crabs), recapitulating the findings of the 2008 survey (Figure 1, site 1). No other points surveyed on Nyakasolo River contained *P. aloysiisabaudiae*. Of special interest was the southern part of the focus in the Mitooma District east of the south Imaramagambo forest reserve. *Potamonautes aloysiisabaudiae* were collected in several sites of tributaries Kamabale and Kanyabiisa of the Nchwera River and further south, on the border with the Rukungiri District in the Nyakyera, a source stream of the Rushaaya. Although in 2008, only single specimen had been collected at one site of the Kamabale (site 3) and the Nyakyera (13), this time *P. aloysiisabaudiae* were collected at several sites of the Kamabale (Figure 2, sites 3–5), of the lower Nchwera and the Butembe, another source stream of the Rushaaya. No crabs were caught in the Nchwera within south Imaramagambo forest. The monthly fly collections at four collection sites established from January 2012 to December 2014 did not result in the capture of a single *S. neavei* fly.

Of the river systems draining to Lake Edwards through Kalinzu, the north Imaramagambo forest reserves and the Queen Elizabeth National Park, Ruzoonga, a tributary of the Nchwera River (Rwempunu), contained a single specimen of *P. aloysiisabaudiae*, which had been collected in 2008. In 2009, no crabs were caught in three sites of the main river or in five sites of the Ruzoonga and its tributaries. No crabs or *S. neavei* were collected on River Nchwera at the edge of the forest reserve even though the habitat seemed to be suitable for both the crab host and the vector.

### Parasitology.

In 2008, skin snip (microscopy) obtained from 294 individuals of all ages in four communities were negative for skin microfilariae by microscopy. In 2009, only five individuals (1.08%), in a total of 462 people from six communities, were positive. All the five individuals were in the age group of 40 years and above (Table 3).

### Serology in 2012.

Analysis of blood spots obtained from a sample of 3,332 children indicated that 13 (0.4%) were positive for Ov16 antibodies, possibly indicating exposure to *O. volvulus*. However, skin snips obtained from all the putative positive children were confirmed negative for parasite DNA by PCR. Thus, all children tested (*N* = 3,332) were confirmed negative for *O. volvulus* infection, resulting in a point prevalence of 0%, within an upper bound of the 95% CI of 0.06%.

### PTS period (2013–2015).

#### Entomological surveys (October 2012–2015).

Consistent with the results obtained before 2012, the results obtained from crab trapping from October 2012 to December 2015, throughout the PTS period ending in December 2015 showed no infestation of crabs (Table 4).

#### Serology in 2015.

A follow-up serosurvey was conducted in 2015 at the end of the PTS period. Blood samples collected from a total of 3,108 children under the age of 10 were screened for the presence of antibodies against Ov16 (Table 5). None of the children screened contained antibodies recognizing Ov16, thus resulting in a point prevalence of 0%, within an upper bound of the 95% CI of 0.06%.

## Discussion

On the basis of entomological, parasitological, and serological data, there appears to be no onchocerciasis transmission in the Imaramagambo focus in 2015, at the end of the PTS period. Only five indigenous adults above 40 years of age (1.1%) were positive for onchocerciasis infection by PCR in skin snips, a confirmation that the area was previously an onchocerciasis-endemic area. However, interruption of transmission of onchocerciasis in this focus cannot be attributed solely to annual mass treatment with ivermectin. Treatment coverage of the eligible persons was generally poor. Indeed, the disappearance of the *S. neavei* flies that were believed to have been the main vector of onchocerciasis in the focus must have hastened the interruption of onchocerciasis transmission and led to its eventual demise.

The freshwater crab *P. aloysiisabaudiae*, which serves as the obligate phoretic host of the larvae and pupae of the vector *S. neavei*, still occurred in some parts of the focus, albeit in low numbers. However, no infested crabs with larval stages of *S. neavei* were observed from 2008 through 2015. This agrees with the anecdotal information from the local people that the crabs, which were common in some areas, gradually became rare or disappeared within recent years.

A decline or disappearance of crabs and *S. neavei* has also been noted in other areas of Uganda. In the Itwara focus, crabs and the vector *S. neavei* have disappeared or become very rare in several of the rivers draining westward into the Rift Valley before any vector control had been performed (J. Katamanywa, E. Mutuuzi, J. Wamani, J. Kashaija, unpublished report). In December 1970, large numbers of crabs, many of them carrying immature *S. neavei*, were trapped in the Nyakibale River (*N* = 153 crabs, and 116 positive) and the Igogonya River (*N* = 54 crabs, and eight positive), according to unpublished records at the Vector Control Unit, Kabarole District. However, in the prospections of 1991, 1993, 1994, and 1995, no crabs were observed in the same rivers in the areas. In 1970, crabs carrying larvae of *S. neavei* were collected in the Dura and Kanyancho rivers in the Kibale forest reserve (now Kibale National Park), but these had disappeared when the same sites were examined in 1991 and 1993.^25^ Another example is the disappearance of *S. neavei* from the Ruteete focus south of Fort Portal in the Kabarole district, where onchocerciasis was hyperendemic in 1971, but is now virtually extinct. In this case, the disappearance of the vector *S. neavei* was explained by the destruction of a forest reserve along the Mahoma River.^25^,^26^ More recently, since about 2000, *S. neavei* and its associated crabs have disappeared from the Nkurungu River, which was regarded as a sub-focus of the Kashoya-Kitomi.^27^ In the Mount Elgon focus of eastern Uganda, the crab population declined substantially both in areas where ground larviciding was applied and where it was never applied.^18^ The decline has been attributed to deforestation and bush clearing resulting from existing subsistence agriculture in the area.

Selective bush clearing was successfully used to eliminate *S. neavei* from one of the former onchocerciasis foci in Kenya.^28^,^29^ However, in the Kibale forest reserve and now Imaramagambo-Kalinzu forest reserves, deforestation is certainly not the reason, as the forests are still intact.

In some cases, like in the Nkurungu River in the Kamwenge District, deforestation and water pollution due to soil erosion after intensive cultivation may have caused the disappearance of the crabs.^29^,^30^ Freshwater crabs prefer to live in rather cool waters and in Uganda have adapted to higher altitudes. It is possible that after deforestation, the rather shallow Nkurungu River warmed and became unsuitable for *Potamonautes*. This certainly cannot explain the absence of crabs from the Kalinzu and Imaramagambo forest reserves, which are still intact. Rivers and streams are still shaded there and resemble those in the Itwara or Kashoya-Kitomi foci. Because most rivers have their sources in open, cultivated, and populated areas east of the forest, pollution cannot be excluded with certainty. One could hypothesize that the large tea plantations, which surround the forests in the northern parts of the focus, might be a source of pollution. A resulting decline of the crab populations could have occurred as the plantations routinely apply herbicides and fertilizers. Yet this does not explain the absence of crabs in rivers in the southern part of the focus, which are far from the tea estates and in areas where agricultural chemicals are not commonly used. On the whole, the decline of the freshwater phoretic host crab population in some parts of Imaramagambo or their disappearance in other parts has resulted in the disappearance of *S. neavei* and the interruption of onchocerciasis transmission.

One could speculate that crab populations were destroyed by a natural catastrophe, for example, a disease among the crabs. A well-documented example of such a disease is the crayfish plague caused by a fungus, the oomycete *Aphanomyces astaci*. This infection virtually wiped out the European crayfish *Astacus fluviatilis* toward the end of the nineteenth century.^31^ The enzootic started around 1860 in northern Italy, reached Central Europe in 1880, and later spread to Russia (1890), Finland (1983), Bulgaria (1900), and Sweden (1907). Viral and bacterial infections are also known from various marine crustaceans. A *Spiroplasma* (Bacteria, Spiroplasmataceae) was identified as the causative agent of tremor disease of the Chinese mitten crab (*Eriocheir sinensis*), which caused mass mortalities in crab populations and became a problem in Chinese aquaculture.^32^ However, nothing similar has been reported from tropical Africa. Interestingly, in the Nchwera and Rushaya systems south of the Imaramagambo focus, none of the female crabs trapped carried eggs or baby crabs, and they were largely immature, with a pleon that was not fully developed. This is a phenomenon usually observed when repopulation of a river system is occurring, and normally after the endemic population was destroyed by unknown factors. Interestingly, the increase in crabs observed from October 2013 to December 2015 was in parts of the rivers far from the tea plantations and the forests. The absence of larval stages of *S. neavei* on these crabs was expected as the flies require shaded conditions that were obviously missing in open rivers.

Based on serological, parasitological, and entomological information obtained from Imaramagambo focus, it was agreed in the 5th annual meeting of UOEEAC held in August 2012 that transmission was no longer occurring in this focus, and recommended that it should be reclassified as “transmission interrupted” and mass drug administration activities ceased so that the focus is moved to a 3-year PTS period. The committee recommended that clinic-based (individual) treatments be provided to those individuals who remain infected with *O. volvulus* within the focus. It is also recommended that entomological and crab assessments continue quarterly throughout the 3-year PTS period. The absence of *S. neavei* flies and infested crabs for a period of slightly over 3 years (January 2012 to February 2015) is a clear indication that onchocerciasis in Imaramagambo has been eliminated, and has not reestablished itself even in the absence of Mass Drug Administration activities.

In conclusion, the data presented above overwhelmingly support the conclusion that there was no autochthonous transmission of onchocerciasis in the Imaramagambo focus for 3 years during the PTS phase. It is thus likely that onchocerciasis has been eliminated from this focus. However, Uganda will have to continue surveillance activities to ensure that the disease is not reintroduced so long as it is endemic in other foci in Uganda and throughout Africa.

## Figures and Tables

**Table 1:** Annual UTG overage (1993–2009)

Year	Total population	UTG	Number treated	% UTG covered
1993	39,780	38,786	12,494	32.2
1994	40,807	39,787	13,678	34.4
1995	41,700	40,658	16,845	41.4
1996	42,856	41,785	21,127	50.6
1997	43,870	42,773	28,405	66.4
1998	45,670	44,528	30,884	69.4
1999	54,497	53,135	28,960	54.5
2000	63,200	61,620	41,831	67.9
2001	65,756	64,112	49,060	76.5
2002	71,514	69,726	50,949	73.1
2003	73,270	71,438	50,873	71.2
2004	74,370	72,511	45,786	63.1
2005	69,686	67,944	38,498	56.7
2006	70,894	69,122	48,409	70.0
2007	82,064	72,580	30,788	42.4
2008	86,480	76,742	0	0.0
2009	102,180	85,748	80,929	94.4

UTG = ultimate treatment goal.

**Table 2:** Sites (cf. Figure 2) in the Imaramagambo onchocerciasis focus where freshwater crabs *Potamonautes aloysiisabaudiae* were collected during surveys conducted in 2008, 2009, and 2012

Site	Date	River system	Crab trapping site	Latitude (S)	Longitude (E)	Crabs trapped	Crabs infested
1	July 24, 2008	Nyakasolo	Nyakasolo bridge	−0.415750	30.21558333	3	0
	July 22, 2012	Nyakasolo	Nyakasolo bridge	−0.415750	30.21558333	3	0
2	July 23, 2008	Nchwera	Ruzoonga	−0.439683	29.9821	1	0
3	July 23, 2008	Kamabale	Kamabale Kagati	−0.535278	29.95163889	1	0
	March 22, 2012	Kamabale	Kamabale Kagati	−0.535278	29.95163889	8	0
	July 25, 2012	Kamabale	Kamabale Kagati	−0.543806	29.95163889	14	0
	May 27, 2012	Kamabale	Kamabale Kagati	−0.535278	29.95163889	18	0
4	January 22, 2012	Kamabale	Kamabale bridge	−0.560472	29.97908333	1	0
5	March 23, 2012	Kamabale	Kamabale lower	−0.543611	29.92730556	14	0
	May 27, 2012	Kamabale	Kamabale lower	−0.543611	29.92730556	5	0
	July 26, 2012	Kamabale	Kamabale lower	−0.543611	29.92730556	11	0
6	July 28, 2012	Nchewera	Kamabale confluence	−0.543611	29.92730556	2	0
7	May 29, 2012	Kanyabisa	Kanyabisa near forest reserve	−0.579417	29.91738889	17	0
8	May 23, 2012	Kanyabisa	Kanyabisa bridge	−0.587694	29.92994444	2	0
9	May 29, 2012	Kanyabisa	Kanyabisa upper	−0.589972	29.93472222	5	0
10	January 22, 2012	Kamabale	Katwe bridge	−0.560472	29.97908333	1	0
	March 21, 2012	Kamabale	Katwe bridge	−0.560472	29.97908333	1	0
	May 23, 2012	Kamabale	Katwe bridge	−0.560472	29.97908333	2	0
	July 24, 2012	Kamabale	Katwe bridge	−0.560472	29.97908333	6	0
	July 30, 2012	Kamabale	Katwe bridge	−0.560472	29.97908333	5	0
	October 1, 2012	Kamabale	Katwe bridge	−0.560472	29.97908333	6	0
11	May 28, 2012	Kamabale	Kamabale upper	−0.563361	29.98227778	3	0
12	July 24, 2012	Butembe	Butembe	−0.651056	29.95886111	1	0
13	May 28, 2012	Nyakyera	Nyakyera bridge	−0.654444	29.94802778	6	0
	July 21, 2008	Nyakyera	Nyakyera bridge	−0.654444	29.94802778	1	0
	July 24, 2012	Nyakyera	Nyakyera bridge	−0.654444	29.94802778	10	0

**Table 3:** Skin snips analyzed by polymerized chain reaction results (2009)

Age group	Number screened	Number positive	% Positive
5–9	29	0	0.0
10–14	78	0	0.0
15–19	52	0	0.0
20–29	91	0	0.0
30–39	74	0	0.0
≥ 40	138	5	3.6
Total	462	5	1.1

**Table 4:** Results from Crab Trapping from October 2012 to December 2015

Year	Month	No. of sites	No. of crabs	
			Caught	Infested
2012	October	19	54	0
2013	January	17	87	0
	April	18	97	0
	July	18	138	0
	October	15	246	0
2014	February	15	162	0
	June	15	120	0
	October	15	173	0
2015	February	16	134	0
	June	16	165	0
	October	16	205	0
	December	19	175	0

**Table 5:** Analysis of blood spots from 3,108 children under 10 years of age in 2015

District	Subcounty	Parish	Children < 10 years screened	Positive	% Positive
Mitooma	Kiyanga	Iraramira	149	0	0
		Kashasha	237	0	0
		Kiyanga	274	0	0
		Rwoburunga	181	0	0
	Kanyambwanga	Kanyabwanga	140	0	0
		Kashongorero	160	0	0
		Rucence	177	0	0
	Bitereko	Bugongo	146	0	0
		Busheregyenyi	184	0	0
		Karimbiro	202	0	0
		Nyakashojwa	149	0	0
	Kashenshero	Bukuba	94	0	0
Bushenyi	Bitooma	Kimuri	127	0	0
		Kashambya	63	0	0
		Bitooma	108	0	0
		Ngorora	95	0	0
	Nyabubare	Nyarugote	102	0	0
	Kyamuhunga	Kabingo	200	0	0
		Mashonga	151	0	0
		Swazi	169	0	0
		Total	3,108	0	0
